# Clinical Outcomes Used in Clinical Pharmacy Intervention Studies in Secondary Care

**DOI:** 10.3390/pharmacy5020028

**Published:** 2017-05-20

**Authors:** Lene Juel Kjeldsen, Charlotte Olesen, Merete Kjær Hansen, Trine Rune Høgh Nielsen

**Affiliations:** 1 The Danish Research Unit for Hospital Pharmacy, Amgros I/S, 2100 Copenhagen, Denmark; 2 The Hospital Pharmacy, Central Denmark Region, 8000 Aarhus, Denmark; CHAOLN@auh.rm.dk; 3 Statistics and Pharmacoepidemiology, Danish Cancer Society Research Center, 2100 Copenhagen, Denmark; mekjha@cancer.dk; 4 Region Zealand Hospital Pharmacy, 4700 Næstved, Denmark; trn@regionsjaelland.dk

**Keywords:** outcomes, clinical pharmacy, hospital, effect, review

## Abstract

The objective was to investigate type, frequency and result of clinical outcomes used in studies to assess the effect of clinical pharmacy interventions in inpatient care. The literature search using Pubmed.gov was performed for the period up to 2013 using the search phrases: “Intervention(s)” and “pharmacist(s)” and “controlled” and “outcome(s)” or “effect(s)”. Primary research studies in English of controlled, clinical pharmacy intervention studies, including outcome evaluation, were selected. Titles, abstracts and full-text papers were assessed individually by two reviewers, and inclusion was determined by consensus. In total, 37 publications were included in the review. The publications presented similar intervention elements but differed in study design. A large variety of outcome measures (135) had been used to evaluate the effect of the interventions; most frequently clinical measures/assessments by physician and health care service use. No apparent pattern was established among primary outcome measures with significant effect in favour of the intervention, but positive effect was most frequently related to studies that included power calculations and sufficient inclusion of patients (73% vs. 25%). This review emphasizes the importance of considering the relevance of outcomes selected to assess clinical pharmacy interventions and the importance of conducting a proper power calculation.

## 1. Introduction

Suboptimal choice of outcomes to assess health care interventions may result in lack of implementation of potentially effective interventions, which could have benefitted the care of patients.

Traditionally, new interventions and services in health care have been implemented if they seemed reasonable, but in recent times with scarce resources, documentation of (cost) effect is essential before implementing a new service. Clinical pharmacy services, including medication reviews, are among many other interventions exposed to documentation of the suggested effect, and indeed, systematic reviews have found some effect of clinical pharmacist interventions in inpatient care [1,2,3,4,5]. However, evaluation of clinical pharmacy services is challenging due to the interventions often being complex and non-specific, and the purpose is often to optimise the use of medications, reduce medication-related risks and improve symptom control [6,7]. Consequently, choice of outcome measures is difficult.

However, choice of outcomes is not the only challenge when conducting outcome research; other essential components include quality of the study, study design, type of intervention, the patient population, etc. [8]. The Donabedian framework is frequently used to evaluate clinical pharmacy services. The model consists of three elements; structure, process and outcome. Structure is the context in which the intervention is delivered, process describes the actions that make up the intervention, and outcomes refers to the effects of the intervention on health status of patients and populations [9,10]. However, most attention is usually given to outcome measures [8,11,12]. 

Outcomes can be categorized into “hard” endpoints, such as mortality and hospital admissions, and “soft” endpoints, such as quality of life, drug-related problems and patient satisfaction. It has been argued that it is essential to select outcomes on which the intervention is likely to have an effect, and that hard endpoints may not be optimal outcome measures, because clinical pharmacy interventions are unlikely to result in changes in these measures [7,8]. In addition, it is essential that a sufficient number of patients are included in the studies (sample size), and a proper power calculation has been performed to ensure knowledge of the minimum number of patients required to detect statistical significance [13]. However, previously no review of the literature has been conducted with the main aim to describe clinical outcomes used in clinical pharmacy intervention studies including the related results reported.

The aim was to investigate type, frequency and result of clinical outcomes used in studies to assess the effect of clinical pharmacy interventions in inpatient care.

## 2. Materials and Methods

### 2.1. Search Strategy

When conducting our literature search, we sought to identify intervention studies performed by clinical pharmacists, which had been evaluated using clinical outcome measures. A literature search was performed using the search phrases: “Intervention(s)” and “pharmacist(s)” and “controlled” and “outcome(s)” or “effect(s)”. 

Publications were included if they:

- described primary research
- were published in English
- described interventions delivered by clinical pharmacists

Publications were excluded if they:

- were not published as a research paper (e.g., reviews, books, congress abstracts, posters, reports, protocols)
- did not include outcome data
- presented data for a secondary study, where the original study had been published previously
- had been conducted in primary care
- included 100 patients or less

The search was performed for the period up to 2013 using PubMed (TRHN).

### 2.2. Assessment

All titles and publication types from the original search were reviewed independently by TRHN and LJK. Subsequently, abstracts were reviewed by the two authors. Thereafter, full-text articles were reviewed independently by CO and LJK. Finally, CO and LJK extracted data form the studies independently. At every step, disagreements were resolved by consensus. The data extracted were details regarding the study, the intervention, outcomes and power calculation.

For each included study, the variable used for power calculation was categorized as “primary outcome” irrespective of whether it was stated to be the “primary outcome” by the authors. Also, when more than one variable was stated to be “primary outcome” by the authors, only variables supported by power calculations were categorized as “primary outcome”. In contrast, if no power calculation was presented and no primary endpoint was stated, all outcomes were categorized as “secondary outcomes” irrespective of the authors stating otherwise.

Some measures were excluded due to assessing qualitative aspects or being descriptive: Number of drugs, drug-related problems (DRPs), acceptance rates, medication knowledge if not assessed using a validated tool, drug burden index, inhalation technique, medication errors unless linked to an event/clinical assessment, drug attitude, quality of well-being, appropriateness of prescribing of individual drugs, self-reported asthma symptoms.

## 3. Results

### 3.1. Study Selection

**Figure 1:**
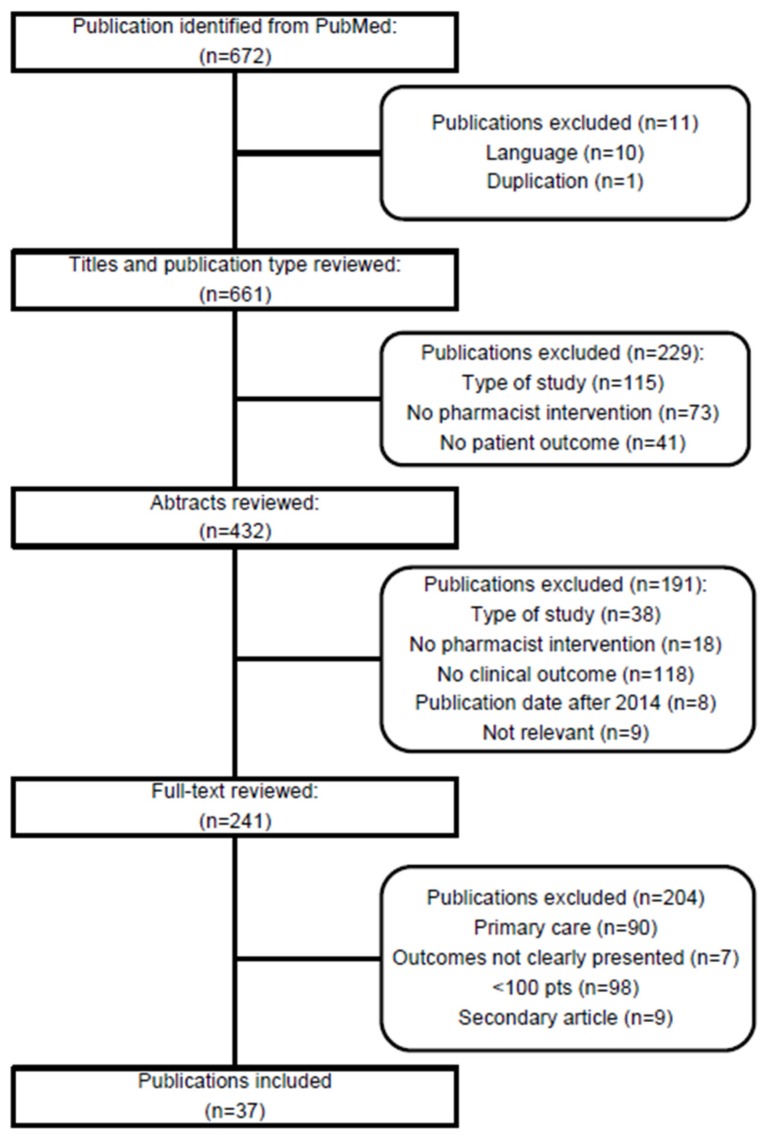
Flow chart of study selection for the review.

A total of 672 studies were identified in the PubMed search (Figure 1). After removing 11 papers due to duplicate publication and non-English language, in- and exclusion criteria were applied to 661 unique publication titles and subsequently to 432 unique abstracts (Figure 1). Of these, 241 full-text publications were reviewed, and 204 were excluded due to: Study conducted in primary care (*n* = 90), outcomes not clearly presented (*n* = 7), ≤100 pts (*n* = 98), and secondary article (*n* = 9). Finally, 37 unique publications were included in the review [14,15,16,17,18,19,20,21,22,23,24,25,26,27,28,29,30,31,32,33,34,35,36,37,38,39,40,41,42,43,44,45,46,47,48,49,50]. Two publications were based on one study, but since different outcome measures were presented in the respective papers, both were included [33,34].

### 3.2. Description of Studies

The included studies had been conducted in 16 countries in Europe, Asia, Australasia, Middle East and North America, and most frequently in the US with ten studies (Table 1). The majority of the studies had been conducted at one hospital (*n* = 30), but four studies included patients from three hospitals and one from 10 hospitals (Table 1). Number of patients included in the study ranged from 105 to 4290 (Table 1). The type of wards and study populations varied considerably, but the majority included patients were suffering from a chronic disease (Table 1).

A traditional randomized, controlled design was applied for the majority (*n* = 26) of the studies (Table 2). The interventions provided appeared similar but differed in types of elements. However, more than half of the studies (*n* = 20) included a combination of patient counselling, medication review and interdisciplinary collaboration (Table 2). Only two studies were finalised with no further follow up at discharge [38,48] (Table 2). All other studies presented interventions which included post-discharge contact with health care professionals or follow-up for effect evaluation—or both—and two studies described interventions with a duration of two years [20,49].

### 3.3. Description of Outcome

The included studies used a plethora (135) of outcome measures to evaluate their interventions ranging from two [15,46] to 13 [14] (Table 3). The most prevalent measures included laboratory measures, clinical measures/assessments by physician and health care service use, however, a large variety of measures within the categories were used. A mixture of generic and disease specific measures was reported (Table 3). Examples of generic measures include medication adherence assessed by the 4-item Morisky Scale, health-related quality of life assessed by SF-36, and service use assessed by LOS in hospital. Examples of disease specific measures comprise knowledge assessed by Malaysian Osteoporosis Knowledge Tool (MOKT), health-related quality of life assessed by QUALEFFO and service use assessed by Number of CHF hospitalizations within 6 months of enrollment.

Some of the studies had selected a primary outcome measure directly related to medication use and knowledge [21,32,34,36,41,44,45,47,50], while others chose measures which may be consequences of the interventions (e.g., laboratory tests, hospital readmission and mortality [14,16,17,18,20,22,23,25,26,27,29,30,31,35,38,40,41,42,43,49]). Adherence, HbA1c values, LDL values, emergency department visits, and hospital readmission were used as primary as well as secondary outcomes.

No apparent pattern was established among primary outcome measures with significant effect in favour of the intervention.

More than half (*n* = 21) of the studies did not present any power calculation (*n* = 13) or did not include sufficient patients according to their power calculation (*n* = 8) (Table 3). Of the 26 primary outcome measures showing a statistically significant effect, 73% reported a power calculation and included sufficient patients according to the power calculation. Only 25% of the 16 primary outcome measures with no statistically significant effect reported a power calculation and included a sufficient number of patients (Table 3).

## 4. Discussion

The literature review included 37 publications worldwide describing quite similar intervention elements but differing in study design. A large variety of outcome measures had been used to evaluate the effect of the interventions; most frequently clinical measures/assessments by physicians and health care service use. No apparent pattern was established among primary outcome measures with significant effect in favour of the intervention, but positive effect was most frequently related to studies that included power calculations and sufficient inclusion of patients.

### 4.1. Outcome Measures

The large variety of outcomes used in the included studies may be explained by the lack of consensus of optimal outcome measures for this type of intervention [11,12].

### 4.2. Generic Versus Disease Specific Tools

Since the interventions are usually complex and the patient populations are often heterogeneous, optimal outcome measures to ensure comparison between studies should be generic. Indeed, numerous generic measures were included in the studies (e.g., adherence measures, ADEs, service use and HRQoL). However, diverging methods were used (e.g., for assessment of adherence (self-reported and objective)), a variety of elements were used (e.g., to assess ADEs (potential and preventable)), different time periods were used (e.g., for assessment of emergency department visits (3 days, 30 days 12 months)) and various tools were used (e.g., for assessment of HRQoL (SF 12, SF 36, self-rated global health)). Even if similar interventions are selected, comparison between the studies would be complicated by differences in type of outcome measure—and design, inclusion criteria, etc.

The large number of disease-specific tools reported as outcome measures may derive from an expectation of these being more relevant for the particular cohort (diversity of patients across studies)—and perhaps an expectation of these measures being more sensitive to change, than generic measures.

Mortality/survival was reported as outcome measures in six studies. The only study providing a power calculation and including sufficient patients showed a positive effect on “Time from randomization to death from any cause” [49]. The continuous variable may be an easier way to evaluate a rare event such as mortality, which usually requires large sample sizes or long follow-up periods to ensure sufficient power [7,8]. However, the aspect of time of follow up is important, since there is a risk of a short follow up resulting in insufficient data (few patients have died) as well as excessive (most patients have died), and this time period is likely to vary according to the characteristics of the included patients. This further complicates the comparison between studies. Hence, survival analysis may be the optimal measure for this outcome. When no effect on an outcome is found in studies with insufficient power, it may be interpreted as “evidence of absence” as in a Cochrane review, while the interpretation should be “absence of evidence” due to lack of power in the included studies [2,51].

### 4.3. Primary Versus Secondary Outcomes

Primary outcomes are used to determine the effect of the intervention, while secondary outcomes evaluate additional effects of the intervention. However, power calculation is only done on primary outcome measures [13]. The number of outcome measures used in the included studies varied considerably (2–13), which may be explained by different needs to determine additional effects of the individual interventions. Laboratory measures, clinical measures/assessments by physician and health care service use were prevalent measures, which may be explained by these measures often being documented as a part of routine patient assessment, and hence easy to collect. Still, they seem to be relevant outcome measures to assess the effect of the studies. 

### 4.4. Target Groups for Results

Another reason for selecting several outcome measures may be the importance of evaluating the intervention with respect to different stakeholders. The importance of an effect may vary according to the perspective, (e.g., patient, care-givers, health care professionals, decision makers and researchers) may not agree on, which outcome measure is the most important [8].

### 4.5. Relevant Outcomes

Further discussions about which outcomes may be relevant to quantify the desired effects of clinical pharmacy interventions are needed. It is important to consider whether an effect can indeed be expected on the selected outcomes [8,11,12]. New approaches to standardize outcome measures in clinical trials are emerging, and the results of this review confirm the need for a standard set of core outcome measures [11,12]. If the aim of clinical pharmacist interventions is to improve symptom control, reduce medication-related risks, improve benefits of medication use and prevent development of conditions, it is possible that outcomes such as preventable adverse drug events, measures directly related to medication use and knowledge, and other soft endpoints are likely to be more appropriate than hard endpoints such as mortality and hospital readmission, since they measure aspects which may be affected by the interventions [8]. A variety of these measures have been used as primary outcome measures in the included studies with varying results.

Finally, it should be kept in mind that even more outcomes may have been used to assess clinical pharmacy interventions, however, a publication bias may exist, which may have led to exclusion of some non-significant or negative outcomes.

### 4.6. Implementation Rate of the Clinical Pharmacy Intervention

Clinical pharmacy interventions usually include provision of professional knowledge to a team of health care professionals or directly to the patient [1,7]. The processes involved when providing knowledge are quite complex, and consequently it is often difficult to measure the pharmacist’s contribution to a multidisciplinary team [8]. Hence, applying process measures as suggested by the Donabedian model is useful to document the tasks actually provided by the clinical pharmacist. Frequently used process measures include type and number of drug-related problems (DRPs) identified, the acceptance rate of suggested recommendations made by the clinical pharmacist to address these DRPs, and implementation rates [1]. However, the acceptance rates and implementation rates of suggested recommendations vary considerably between studies, with usually around 65–70% acceptance rates—but some as low as 40% [1,2]. Whether low acceptance and implementation rates are due to suboptimal recommendations, barriers among physicians to accept and implement recommendations, or poor collaboration in the health care team remains unclear, and no suggestions of a minimum requirement for acceptance or implementation rates exist. This pose another challenge of interpreting outcomes, since studies with a sufficient number of included patients may not have had a proper exposure of the intervention to intervention patients. Consequently, the success of the clinical pharmacy intervention may be highly dependent on individual participants in the health care team, including the clinical pharmacist herself.

### 4.7. Limitation

Various methods exist to assess the quality of intervention studies (e.g., criteria developed by the Cochrane Effective Practice and Organisation of Care Review Group [52]). No formal quality assessment of the included studies was performed in the present review due to the exploratory nature of the review, however, ensuring sufficient power in a study is essential to avoid Type II errors, and more than half of the studies either did not include sufficient patients according to their power calculation or the power calculation was missing. This risk of Type II errors complicates the assessment of the potential effect and relevance of the selected outcome variables [13].

Types of statistical analyses used were not systematically collected. Comparison between studies may be further compromised, when different analyses are used i.e., continued variables (linear regression and ANOVA), binary outcomes (logistic regression), time to event (survival analysis), etc., since type of analysis is important for interpretation of the results.

Other aspect regarding the analyses, which was not systematically collected, were handling of dropouts and incomplete data (e.g., “last observation carried forward”, exclusion, imputation, etc.) These may also affect the results and hence the interpretation of results differently.

Further, studies including 100 patients or less were excluded. It is likely that if they had been included, the proportion of studies with no reported power calculation and insufficient power may have been higher.

## 5. Conclusions

Type, frequency and result of clinical outcomes used to assess the effect of clinical pharmacy interventions in inpatient care varied considerably among the included studies. The most frequently reported outcome measures included clinical measures/assessments by physician and health care service use. No obvious pattern was established among primary outcome measures with significant effect in favour of the intervention, but positive effect was most frequently related to studies with presentation of power calculations and sufficient inclusion of patients. This review emphasizes the importance of considering the relevance of outcomes selected to assess clinical pharmacy interventions. Further discussion and consensus is needed with regard to selection of types of outcomes to ensure comparison of the effects among clinical pharmacy studies. Furthermore, conducting a proper power calculation and including the sufficient number of patients in the study according to the power calculation should be a prerequisite when publishing an outcome evaluation of clinical pharmacy intervention studies.

## Figures and Tables

**Table 1:** Description of the studies.

Author	Setting and Country	Patient Population	No. of Included Patients	No. of Patients Analysed/at Endpoint	Mean Age (Years) IG	Mean Age (Years) CG	Gender, Male (%) IG	Gender, Male (%) CG
Al Mazroui et al. (2009) [14]	General medical wards, endocrinology and medical outpatient clinics, 1 Hospital, UAE	Pts with type 2 diabetes	240 pts: IG: 120 pts CG: 120 pts	234 pts: IG: 117 CG: 117	48.7, *n* = 120	49.9, *n* = 120	84 (70), *n* = 120	82 (68.3), *n* = 120
Albsoul-Younes et al. (2011) [15]	1 family medicine clinic, 1 hospital, Jordan	Pts with uncontrolled hypertension	266 pts: IG: 136 pts CG: 130 pts	253 pts: IG: 130 pts CG: 123	56.3, *n* = 130	57.5, *n* = 123	61 (47), *n* = 130	59 (48), *n* = 123
Barker et al. (2012) [16]	1 hospital, Australia	Pts with chronic heart failure	120 pts: IG = 64 pts CG = 56 pts	87 pts: IG: 48 pts CG: 39 pts	73.0, *n* = 64	72.0, *n* = 56	32 (50), *n* = 64	23 (41), *n* = 56
Bladh et al. (2011) [17]	2 internal medicine wards, 1 hospital, Sweden	All patients admitted to the wards on week days	400 pts: IG: 199 pts CG: 201 pts	345 pts: IG: 164 CG: 181	Median: ITT: 81, *n* = 164 PP: 84, *n* = 87	Median: ITT/PP: 82, *n* = 181	ITT: 66 (40), *n* = 164 PP: 30 (34), *n* = 87	ITT/PP: 71 (39), *n* = 181
Chan et al. (2012) [18]	1 diabetics clinic, 1 hospital, Hong Kong	Pts with type 2 diabetes	105 pts: IG: 51 pts CG: 54 pts	105 pts: IG: 51 pts CG: 54 pts	63.2, *n* = 51	61.7, *n* = 54	30 (59), *n* = 51	28 (52), *n* = 54
Chiu et al. (2008) [19]	Outpatients, 1 hospital, Taiwan	Pts with ischemic stroke	160 pts: IG: 80 pts CG: 80 pts	Missing	65.7, *n* = 80	64.8, *n* = 80	40 (50), *n* = 80	40 (50), *n* = 80
Chung et al. (2011) [20]	1 lipid clinic (medical outpatient), 1 hospital, Hong Kong	Pts with chronic dyslipidaemia	300 pts: IG: 150 pts CG: 150 pts	300 pts: IG: 150 pts CG: 150 pts	56.2, *n* = 150	57.9, *n* = 150	68 (45), *n* = 150	60 (40), *n* = 150
Crotty et al. (2004) [21]	3 hospitals, Australia	Elderly pts awaiting transfer from hospital to a long term residential care facility for the first time	110 pts: IG: 56 pts CG: 54 pts	88 pts: IG: 44 pts CG: 44	82.0	83.4	41%	37%
Dedhia et al. (2009) [22]	General medicine wards, 3 hospitals, USA	Pts aged ≥65 years	422 pts: IG: 185 pts CG: 237 pts	422 pts: IG: 185 pts CG: 237 pts	76.7	77.3	72 (39), *n* = 185	94 (40), *n* = 237
Gillespie et al. (2009) [23]	2 acute internal medicine wards, 1 hospital, Sweden	Pts admitted to the wards	400 pts: IG: 199 pts CG: 201 pts	368 pts: IG: 182 pts CG: 186 pts	86.4, *n* = 182	87.1, *n* = 186	77 (42), *n* = 182	75 (40) *n* = 186
Hammad et al. (2011) [24]	6 family medicine outpatient clinics, 1 Hospital, Jordan	Pts with metabolic syndrome	202 pts: IG: 112 pts CG: 90 pts	199 pts: IG: 110 pt CG: 89 pts	56.0, *n* = 110	57.4, *n* = 89	44 (40), *n* = 110	32 (36), *n* = 89
Hellström et al. (2012) [25]	3 internal medicine wards, 1 hospital, Sweden	All patients hospitalised at the three study wards	4290 pts: IG: 1325 CG: 2965	3974 pts: IG: 1216 pts CG: 2758	78.3	79.5	46%	45%
Jack et al. (2009) [26]	1 hospital, USA (entire hospital)	Pts admitted to the hospital, ≥18 years and English speaking	749 pts: IG: 373 pts CG: 376 pts	738 pts: IG: 370 pts CG: 368 pts	50.1, *n* = 373	49.6, *n* = 376	195 (52), *n* = 373	176 (47), *n* = 376
Jackson et al. (2004) [27]	1 hospital, Australia (entire hospital)	Pts initiated on warfarin in hospital	128 pts: IG: 60 pts CG: 68 pts	127 pts: IG: 59 pts CG: 68 pts	Median: 70, *n* = 60	Median: 72.5, *n* = 68	53%, *n* = 60	53%, *n* = 68
Jacobs et al. (2012) [28]	An ambulatory general internal medicine setting, 1 Clinic, USA	Pts with type 2 diabetes	396 pts: IG: 195 pts CG: 201 pts	164 pts: IG: 72 pts CG: 92 pts	62.7, *n* = 72	63.0, *n* = 92	49 (68), *n* = 72	51 (55), *n* = 92
Jarab et al. (2012a) [29]	1 outpatient COPD Clinic, 1 Hospital, Jordan	Pts with COPD	133 pts: IG: 66 pts CG: 67 pts	127 pts: IG: 63 pts CG: 64 pts	Median: 61, *n* = 66	Median: 64, *n* = 67	26 (39), *n* = 66	28 (42), *n* = 67
Jarab et al. (2012b) [30]	outpatient diabetes clinic, 1 hospital, Jordan	Pts with type 2 diabetes	171 pts: IG: 85 pts CG: 86 pts	IG: 77 pts, CG: 79 pts	63.4, *n* = 85	65.3, *n* = 86	68%, *n* = 85	56%, *n* = 86
Kirwin et al. (2010) [31]	1 hospital-based, primary care practice, 1 hospital, USA	Pts with diabetes (type 1 and 2)	346 pts: IG: 171 pts CG: 175 pts	301 pts: IG: 150 pts CG: 151 pts	62.9, *n* = 150	62.8, *n* = 151	29% *n* = 150	39% *n* = 151
Kripalani et al. (2012) [32]	2 medical centers, 2 hospitals, USA	Pts with acute coronary syndromes or acute decompensated heart failure	862 pts: IG: 430 pts CG: 432 pts	851 pts: IG: 423 pts CG: 428 pts	61, *n* = 423	59, *n* = 428	250 (59), *n* = 423	249 (58), *n* = 428
Lai et al. (2013) [33]	1 osteoporosis clinic, 1 hospital, Malaysia	Pts with postmenopausal osteoporosis	198 pts: IG: 100 pts CG: 98 pts	177 IG:88 pts CG: 89 pts	65.1, *n* = 100	67.1, *n* = 98	Missing	Missing
Lai et al. (2011) [34]	1 osteoporosis clinic, 1 hospital, Malaysia	Pts with postmenopausal osteoporosis	198 pts: IG: 100 pts CG: 98 pts	177 IG:88 pts CG: 89 pts	65.1, *n* = 100	67.1, *n* = 98	Missing	Missing
Lee et al. (2009) [35]	3 Out-Patient Departments, 3 hospitals, Hong Kong	Pts with hyperlipidaemia	119 pts: IG: 59 pts CG: 60 pts	118 pts: IG: 58 pts CG: 60 pts	63, *n* = 58	61, *n* = 60	34 (59), *n* = 58	26 (43), *n* = 60
Lim et al. (2004) [36]	1 geriatric outpatient clinic, 1 hospital, Singapore	Elderly outpatients with risk factors of non-compliance	136 pts: IG: 68 pts CG: 68 pts	126 pts IG: 64 pts CG: 62 pts	79.6, *n* = 64	80.5, *n* = 62	39%, *n* = 64	31%, *n* = 62
Magid et al. (2011) [37]	3 healthcare systems, USA	Pts with uncontrolled BP	338 pts: IG: 174 pts CG: 164 pts	283 pts IG: 138 pts CG: 145 pts	65.1, *n* = 138	66.7, *n* = 145	67%, *n* = 138	63%, *n* = 145
McCoy et al. (2012) [38]	1 hospital, USA (entire hospital)	Pts with an acute 0.5 mg/dL change in serum creatinine over 48 h and a nephrotoxic or renally cleared medication order	540 pts: IG: 262 pts CG: 278 pts	396 pts IG: 200 pts CG: 196 pts	60.7, *n* = 200	58.3, *n* = 196	53%, *n* = 200	61%, *n* = 196
Mergenhagen et al. (2012) [39]	2 general medical units, 1 hospital, USA (entire hospital)	Pts admitted for at least 24 h to one of the study units	359 ams: 111 ams (pharmacist) 248 ams (physician)	218 ams: 102 ams (pharmacist) 116 ams (physician)	PharmG: 68, *n* = 102	PhysG: 68, *n* = 116	PharmG: 100%, *n* = 102	PhysG: 98%, *N* = 116
Morgado (2011) [40]	1 hospital care hypertension/dyslipidemia outpatient clinic, 1 hospital, Portugal	Pts with essential hypertension	197 pts: IG: 98 pts CG: 99 pts	Missing	58.3, *n* = 99	60.7, *n* = 98	44 (45), *n* = 99	35 (35), *n* = 98
Murray et al. (2007) [41]	1 ambulatory care practice, USA	Pts with heart failure, low-income, ≥50 years	314 pts: IG: 122 pts CG: 192 pts	270 pts: IG: 106 pts CG: 164 pts	61.4, *n* = 122	62.6, *n* = 192	39 (32), *n* = 122	65 (34), *n* = 192
Sadik et al. (2005) [42]	General medical wards, cardiology and medical outpatient clinics, 1 hospital, UAE	Pts with heart failure	221 pts IG: 109 pts CG: 112 pts	208 pts IG: 104 pts CG: 104 pts	58.6, *n* = 104	58.7, *n* = 104	52 (50), *n* = 104	52 (50), *n* = 104
Schnipper et al. (2006) [43]	General medicine service, 1 hospital, USA	Pts discharged home	178 pts: IG: 92 pts CG: 84 pts	IG: 79, CG: 73 pts	60.7, *n* = 92	57.7, *n* = 84	33%, *n* = 92	35%, *n* = 84
Spinewine et al. (2007) [44]	1 acute Geriatric Evaluation and Management (GEM) unit, 1 hospital, Belgium	Pts aged ≥70 years	203 pts	186 pts IG: 96 pts CG: 90 pts	82.4, *n* = 96	81.9, *n* = 90	28%, *n* = 96	33%, *n* = 90
Stange et al. (2013) [45]	1 medical Center, 1 hospital, Germany	Pts with chronic hypertension, diabetes, and/or dyslipidemia	240 pts IG: 132 pts CG: 108 pts	162 pts: IG:89 pts CG: 73 pts	64.4, *n* = 129	63.2, *n* = 108	81 (63), *n* = 129	90 (83), *n* = 108
Suppapitiporn et al. (2005) [46]	1 endocrine Clinic, 1 hospital, Thailand	Pts with type 2 diabetes	360 pts: IG: 180 IG 1 = 50 pts IG 2 = 50 pts IG 3 = 30 pts IG 4 = 50 pts CG: 180	Missing	61.4, *n* = 180	59.9, *n* = 180	59 (33), (*n* = 180)	64 (36), *n* = 180
Tsuyuki et al. (2004) [47]	10 hospitals, Canada	Pts with heart failure	276 pts: IG: 140 pts CG: 136 pts	Missing	71, *n* = 140	72, *n* = 136	81 (58), *n* = 140	79 (58), *n* = 136
von Gunten et al. (2005) [48]	General medical wards and intensive care units, 3 hospitals, Switzerland	Pts receiving antibiotic treatment	1200 pts: IG; 600 pts, CG: 600 pts IG1: 200 + 200 pts IG2: 200 + 200 pts CG: 200 + 200 pts	Missing	Different categories	Different categories	Different categories	Different categories
Wu et al. (2006) [49]	Specialist medical clinics, 1 hospital, Hong Kong	Non-compliant pts with polypharmacy	442 pts: IG: 219 pts CG:223 pts	Missing	71.2, *n* = 219	70.5, *n* = 223	108 (49), *n* = 219	107 (48), *n* = 223
Zhang et al. (2012) [50]	1 pediatric unit, 1 hospital, China	Pediatric pts with nerve system disease, respiratory system disease or digestive system disease	160 pts: IG: 80 pts CG: 80 pts	150 pts: IG: 76 pts CG: 74 pts	Age groups	Age groups	43 (54), *n* = 80	44 (55), *n* = 80

IG = Intervention group, CG = Control group.

**Table 2:** Description of study designs and intervention elements used in the included studies.

Author	Intervention Elements							Study Design	Duration of Study (Intervention Period)/Monitoring	Post Intervention Follow-up
	Patient counselling/education *	Adherence assessment/intervention	Medication reconciliation	Medication review	Interdisciplinary collaboration in hospital	Therapeutic drug monitoring	Collaboration between primary acare and inpatient care			
Al Mazroui et al. (2009) [14]	X			X	X			RCT	Visits at 4 months, 8 months and 12 months	No further follow-up
Albsoul-Younes et al. (2011) [15]	X	X		X	X			RCT	Regular monthly visits to the clinic during 6 months	No further follow-up
Barker et al. (2012) [16]	X	X		X	X		X	RCT	Home visits within 96 h of discharge, at 1 and 6 months	No further follow-up
Bladh et al. (2011) [17]	X			X	X		X	RCT		6-month follow-up
Chan et al. (2012) [18]	X	X		X	X			RCT	Intervention delivered at each clinic visit during 9 months after enrolment	No further follow-up
Chiu et al. (2008) [19]	X **	X						Stratified RCT	The intervention was delivered monthly during 6 months	No further follow-up
Chung et al. (2011) [20]	X	X		X	X			Prospective controlled trial	3 clinic visits and monthly telephone follow-ups during 24 months	No further follow-up
Crotty et al. (2004) [21]				X			X	RCT	1 interdisciplinary, cross-sectorial meeting at the long term care facility 14–28 days after discharge	8-week follow-up
Dedhia et al. (2009) [22]			X	X	X		X	Quasi-experimental pre–post study design	.	1-week and 30-day follow-up
Gillespie et al. (2009) [23]	X			X	X		X	RCT	1 follow-up telephone 2 months after discharge	12-month follow-up
Hammad et al. (2011) [24]	X	X		X	X			RCT	The intervention was delivered monthly during 6 months	No further follow-up
Hellström et al. (2012) [25]			X	X	X		X	Prospective, controlled study	.	6-month follow-up
Jack et al. (2009) [26]			X	X	X		X	RCT	1 follow-up phone call by clinical pharmacist 2 to 4 days after discharge	30-day follow-up
Jackson et al. (2004) [27]	X					X	X	Open-label RCT	4 home visits by clinical pharmacist on alternate days after discharge	90-day follow-up
Jacobs et al. (2012) [28]	X			X	X		X	Prospective, randomized, clinical practice study		12-month follow-up
Jarab et al. (2012a) [29]	X	X						RCT		6-month follow-up
Jarab et al. (2012b) [30]	X			X	X			RCT	8-week telephone follow-up call by clinical pharmacist	6-month follow-up
Kirwin et al. (2010) [31]				X			X	RCT		30-day follow-up
Kripalani et al. (2012) [32]	X	X	X	X	X		X	RCT	1 telephone follow-up 1-4 days after discharge	30-day follow-up
Lai et al. (2013) [33]	X	X		X				RCT	Monthly follow-up via telephone calls for the first 6 months, then every 3 months until month 12	No further follow-up
Lai et al. (2011) [34]	X	X		X				RCT	Monthly follow-up via telephone calls for the first 6 months, then every 3 months until month 12	No further follow-up
Lee et al. (2009) [35]	X	X		X	X			RCT	A telephone follow-up every 4 weeks and a follow-up interview on the date of the following physician visit within 16 weeks.	No further follow-up
Lim et al. (2004) [36]	X	X		X	X			RCT		2-month follow-up
Magid et al. (2011) [37]	X	X		X	X		X	RCT	6-month follow-up	No further follow-up
McCoy et al. (2012) [38]				X	X			Randomized clinical trial		No follow-up
Mergenhagen et al. (2012) [39]			X					Quasi-experimental study. Subgroup analysis of a prospective, nonrandom, analytic cohort study with concurrent controls		1-month follow-up
Morgado (2011) [40]	X			X	X			RCT	3, 6 and 9-month follow-up	No further follow-up
Murray et al. (2007) [41]	X			X	X		X	RCT	A pharmacist provided a 9-month multilevel intervention	3-month follow-up
Sadik et al. (2005) [42]	X			X	X		X	RCT	Clinic visits at 3, 6, 9 and 12 months	No further follow-up
Schnipper et al. (2006) [43]	X	X	X	X	X		X	RCT	A follow-up telephone call 3 to 5 days after discharge	30-day follow-up
Spinewine et al. (2007) [44]	X			X	X		X	RCT		1 month, 3 months, and 1 year follow-up
Stange et al. (2013) [45]				X	X		X	Prospective, semi-randomized study		6-week follow-up
Suppapitiporn et al. (2005) [46]	X	X						RCT	Follow-up visits at 3 and 6 months	No further follow-up
Tsuyuki et al. (2004) [47]	X	X						Mixed design - partly RCT: Stage 1: In-hospital intervention in all patients Stage 2: randomized trial.	Follow-up at 2 weeks, 4 weeks, then monthly for 6 months after discharge	No further follow-up
von Gunten et al. (2005) [48]				X	X			Pre-post study. Randomised at hospital level		No follow-up
Wu et al. (2006) [49]	X	X						RCT	6-8 telephone calls and a finalizing visit during a 2-year follow-up	No further follow-up
Zhang et al. (2012) [50]	X			X	X			RCT	Patients were usually interviewed on phone when discharge drugs were half finished	2-week follow-up

* Patient counselling/education covers a large variety of activities including discharge counselling, patient education regarding medication and lifestyle etc. These activities are, however, often vaguely described and are consequently difficult to further categorise. ** Group education of patients.

**Table 3:** Outcome measures used in the included studies. The numbers in the cells are reference numbers.

Measure	Primary Outcome		Secondary Outcome		Total
	Statistical Difference in Favour of Intervention	No Statistical Difference in Favour of Intervention	Statistical Difference in Favour of Intervention	No Statistical Difference in Favour of Intervention	
**Medication regimen characteristics**					
Unnecessary drug use				44	1
Duration of antibiotic treatment				48	1
Composite score (dose, frequency and indication)			36		1
Unplanned cessation of warfarin				27	1
Medication regimen intensity			37		1
Medication complexity	45 ^B^				1
Drug specific quality indicators				17	1
72-h medication-prescribing risk score				39	1
Medication appropriateness index (MAI)	19, 44				2
Beers criteria		44			1
Assessing Care of Vulnerable Elders (ACOVE) underuse	44				1
Medication discrepancies				43	1
The number of clinically important medication errors per patient during the first 30 days after hospital discharge		32			1
Time to provider modification or discontinuation of targeted nephrotoxic or renally cleared medications				38	1
Medication beliefs			29		1
**Adherence to medication**					
Medication adherence/compliance self-reported (no validated tool)	50		14, 36, 40, 42		5
Medication adherence/compliance self-reported “Medication Adherence Rating Scale” (MARS-D)		45 ^B^			1
Medication adherence/compliance self-reported (4-item Morisky Scale)			29, 30		2
Medication adherence/compliance objectively assessed	41	47	18	37	4
Medication adherence/compliance self-reported and objectively assessed	34 ^A^		49	43	3
Persistence		34 ^A^			1
**Adherence to guidelines**					
British National Formulary			14		1
Lifestyle advice adherence			14, 42		2
Adherence to guidelines				48	1
Adherence to screening for retinopathy, neropathy, and microalbuminuria			28		1
Annual (LDL-C) testing				31	1
Annual urine microalbumin testing				31	1
Rates of pneumococcal vaccination				31	1
Change in rates of semiannual A1c testing from baseline to 30-day follow-up		31 ^B^			1
Frequency of primary care providers’ follow-up within 30 days of discharge			26		1
Annual eye exam			31		1
**Adverse drug events/reactions**					
ADE (total)			39	21, 43	3
Potential adverse drug events				32	1
Potential Acute kidney injury (AKI) ADEs		38 ^A^			1
Acute kidney injury (AKI) related ADEs		38 ^A^			1
Preventable ADEs	43 ^B^				1
ADEs from admission prescribing errors			39		1
Clinically important ADEs				32	1
Adverse drug reactions				50	1
Residual ADRs at month 2			36		1
**Laboratory measures**					
HbA1c	14, 30 ^B^		18, 28, 46	19, 31	7
Fasting blood glucose			30, 46	19, 24	4
Postprandial blood glucose				19	1
Total cholesterol			14, 20, 30, 35	19	5
HDL			14, 35	18, 20, 24, 30	6
LDL	35 ^B^		14, 18, 19, 20, 28, 30	31	8
Triglycerides			14, 19, 20, 24, 30, 35	18	7
The achievement of a therapeutic INR value on day 8 after discharge	27				1
% patients achieving the ATP III LCL-C goal at the end of the study	20				1
Urinary albumin-to-creatinine ratio (ACR)				18	1
**Clinical measures/assessment by physicians**					
BP			14, 15, 19, 24, 30	18, 31, 42	8
Systolic BP	40			28	2
Diastolic BP			28, 40		2
BP control			40		1
Achieving BP goals			15	37	2
Pulse				42	1
Waist circumference				24	1
Body weight				24, 42	2
BMI			14	18, 30	3
Symptoms				42	1
Bone turnover markers (BTMs)		34 ^A^			1
Clinical status according to primary physician				36	1
2-min walk test			42		1
Forced vital capacity (FVC) measured by spirometer			42		1
Bleeding events 3 months after discharge	27				1
Falls				21	1
Framingham prediction scores			14		1
Change in coronary heart disease (CHD) risk	18				1
Changes in stroke risk			18		1
Shift from a status of MS to no MS			24		1
Worsening mobility				21	1
Worsening behaviours				21	1
Increased confusion				21	1
Worsening pain			21		1
**Resource utilization**					
Length of stay (LOS) in hospital			47, 49, 50	48	4
*Cardiovascular-related* LOS			47		1
Physician visits				47	1
*Cardiovascular-related* Physician visits				47	1
Emergency department visits/casual department visits	23			47, 49	3
Emergency department visits (within 3 days)			22		1
Emergency department visits (within 30 days)			22		1
Emergency visits up to 12 months after discharge				44	1
*Cardiovascular-related* Emergency room visits			47		1
Time to emergency department revisits after discharge		25 ^A^			1
Hospital readmission/hospital admission	23		49	44, 47, 50	6
30 day readmission rate	22 ^B^				1
Drug-related readmissions	23				1
Unplanned readmission				27	1
*Cardiovascular-related* Hospital readmissions				47	1
Readmissions to hospital due to anticoagulant-related complications within 90 days of initial discharge		27			1
Number of all cause and CHF hospitalization within 6 months of enrolment		16 ^A^			1
Number of CHF hospitalization within 6 months of enrolment		16 ^A^			1
Days of all cause and CHF hospitalization within 6 months of enrolment		16 ^A,C^			1
Days of non-CHF-hospitalization within 6 months of enrolment			16		1
Combination of emergency department visits and hospital readmissions			21		1
Emergency department visits and hospitalizations within 30 days of discharge	26				1
Preventable medication related emergency department visits or readmissions			43		1
Exacerbations requiring emergency department care or hospital admission	41				1
The combined rate of post-discharge hospital revisits or death (ED visit, hospitalization or death)				25	1
Health care utilization (scheduled and unscheduled office visits, urgent care and ED visits, and hospital admissions)				43	1
Costs					
Costs			23, 26, 47		3
Total direct costs			41		1
Cost of antibiotic treatment				48	1
Cost of drugs and hospitalization				50	1
*Cardiovascular-related* Cost			47		1
Cost-effectiveness			18		1
Cost avoidance			36		1
Mortality					
Mortality (general)				23, 27, 44	3
Mortality within 6 months of enrolment		16 ^A^			1
Time from randomisation to death from any causes	49				1
Event-free survival				25	1
Quality of Life/Health related quality of life					
Short form 36 (SF 36)			14, 16, 42	16, 42	5
Short form 12 (SF 12)				45	1
EuroQol 5 dimension (EQ-5D)		17 ^B^			1
Self-rated global health			17	17	2
Assessment of quality of life (AQoL)				16	1
Minnesota living with heart failure questionnaire (MLHF)	42				1
St George Respiratory Questionnaire (SGRQ)		29 ^B^			1
Chronic Heart Failure Questionnaire				41	1
Quality of Life Questionnaire of the European Foundation for Osteoporosis (QUALEFFO)			33		1
Patient knowledge					
Patient medication knowledge	36		14, 18	42	4
COPD knowledge			29		1
Patients’ knowledge of target BP values and of hypertension risks			40		1
Malaysian Osteoporosis Knowledge Tool (MOKT)			33		1
Satisfaction and perception					
Satisfaction with information about medications				44, 45	2
Patient satisfaction with pharmacy services			41		1
Osteoporosis Patient Satisfaction Questionnaire (OPSQ)			33		1
Satisfaction with hospitalization and discharge processes				43	1
Coleman’s Care Transition Measures			22		1
Patient perception (perception of severity of illness, usefulness of treatment and appropriateness of the number of medications)				36	1
Other					
Self-perceived health status			22		1
Identification of index discharge diagnosis			26		1
Identification of primary care provider name			26		1
Self-reported preparedness for discharge			26		1
Self-care activities (Diabetes Self-Care Activities questionnaire)			30		1
Total	26	16	96	78	216

^A^: Sample size calculation missing for: 15, 16, 19, 24, 25, 28, 33, 34, 37, 38, 39, 46, 48; ^B^: Sample size not achieved for: 17, 22, 29, 30, 31, 35, 43, 45; ^C^: Difference in favour of control group.
